# The *Arabidopsis* Transcription Factor ANAC032 Represses Anthocyanin Biosynthesis in Response to High Sucrose and Oxidative and Abiotic Stresses

**DOI:** 10.3389/fpls.2016.01548

**Published:** 2016-10-14

**Authors:** Kashif Mahmood, Zhenhua Xu, Ashraf El-Kereamy, José A. Casaretto, Steven J. Rothstein

**Affiliations:** Department of Molecular and Cellular Biology, University of Guelph, GuelphON, Canada

**Keywords:** ANAC032, anthocyanin biosynthesis, *Arabidopsis thaliana*, high light, salinity, oxidative stress

## Abstract

Production of anthocyanins is one of the adaptive responses employed by plants during stress conditions. During stress, anthocyanin biosynthesis is mainly regulated at the transcriptional level via a complex interplay between activators and repressors of anthocyanin biosynthesis genes. In this study, we investigated the role of a NAC transcription factor, ANAC032, in the regulation of anthocyanin biosynthesis during stress conditions. *ANAC032* expression was found to be induced by exogenous sucrose as well as high light (HL) stress. Using biochemical, molecular and transgenic approaches, we show that ANAC032 represses anthocyanin biosynthesis in response to sucrose treatment, HL and oxidative stress. ANAC032 was found to negatively affect anthocyanin accumulation and the expression of anthocyanin biosynthesis (*DFR, ANS/LDOX) and* positive regulatory (*TT8)* genes as demonstrated in overexpression line (35S:ANAC032) compared to wild-type under HL stress. The chimeric repressor line (35S:ANAC032-SRDX) exhibited the opposite expression patterns for these genes. The negative impact of ANAC032 on the expression of *DFR, ANS/LDOX* and *TT8* was found to be correlated with the altered expression of negative regulators of anthocyanin biosynthesis, *AtMYBL2* and *SPL9*. In addition to this, ANAC032 also repressed the MeJA- and ABA-induced anthocyanin biosynthesis. As a result, transgenic lines overexpressing *ANAC032* (35S:ANAC032) produced drastically reduced levels of anthocyanin pigment compared to wild-type when challenged with salinity stress. However, transgenic chimeric repressor lines (35S:ANAC032-SRDX) exhibited the opposite phenotype. Our results suggest that ANAC032 functions as a negative regulator of anthocyanin biosynthesis in *Arabidopsis thaliana* during stress conditions.

## Introduction

Anthocyanin pigments belong to the flavonoid-class of secondary metabolites originating from the phenylpropanoid pathway. Production of anthocyanins is spatio-temporally regulated in different plant species (Jackson et al., 1992; Procissi et al., 1997; Gou et al., 2011). In addition, anthocyanin biosynthesis is also induced in response to a variety of stress conditions such as UV light, HL (Vanderauwera et al., 2005; Cominelli et al., 2008; Morishita et al., 2009; Lotkowska et al., 2015; Viola et al., 2016), nutrient deficiency (Jiang et al., 2007; Peng et al., 2008), drought (Castellarin et al., 2007), salinity (Eryilmaz, 2006; Lotkowska et al., 2015) and extreme temperature fluctuations (Christie et al., 1994; Yamane et al., 2006). Under HL and UV irradiation, anthocyanins act as a sun screen and function in protecting photosynthetic organs from photoinhibition (Hughes et al., 2005; Albert et al., 2009). Other abiotic stresses such as drought, salinity and extreme temperature fluctuations also result in ROS production, and anthocyanin accumulation during these conditions is likely to confer tolerance through ROS scavenging (Nakabayashi et al., 2014).

The enzymatic pathway involved in the production of anthocyanin pigments has been well elucidated with the identification of a number of mutants in different plant species, consisting of early and late biosynthesis genes (EBGs and LBGs). EBGs such as *chalcone synthase* (*CHS*), *chalcone isomerase* (*CHI*), *flavanone 3-hydroxylase* (*F3H*), and *flavonoid 3′-hydroxylase* (*F3′H*) are involved in the production of different types of flavonoids including anthocyanin. LBGs such as *dihydroflavonol 4-reductase* (*DFR*), *leucoanthocyanidin oxygenase* (*LDOX*), *anthocyanidin reductase* (*ANR*) and *UDP-glucose:flavonoid 3-O-glucosyltransferase* (*UF3GT*) are specific for anthocyanin biosynthesis only. The regulation of anthocyanin production is mainly achieved through a concerted action of a number of transcription factors (TFs). Both positive and negative transcriptional regulatory mechanisms for anthocyanin production have been identified. Positive transcriptional regulation in *Arabidopsis thaliana* involves the formation of a MBW protein complex, comprising members of the MYBs (TT2, PAP1; MYB75, PAP2, MYB113 or MYB114), bHLHs (TT8, GL3 and EGL3) and WD40-repeat proteins (TTG1) (Walker et al., 1999; Carey et al., 2004; Teng et al., 2005; Baudry et al., 2006; Quattrocchio et al., 2006; Gonzalez et al., 2008). In the context of negative regulation, two members of the small R3-MYB TF family have been identified in *Arabidopsis* to repress anthocyanin biosynthesis. One is AtMYBL2 that interacts with TT8 to form the L2BW complex which represses anthocyanin biosynthesis (Dubos et al., 2008; Matsui et al., 2008). Interplay between MBW and L2BW complex has thus been proposed to regulate anthocyanin biosynthesis in *A. thaliana* (Dubos et al., 2008; Matsui et al., 2008). Another small R3-MYb protein, CPC, has been implicated to repress anthocyanin biosynthesis by interacting with PAP1/PAP2 and interfering with the formation of the MBW complex (Zhu et al., 2009; Nemie-feyissa et al., 2014). In addition to R3-MYBs, a SQUAMOSA PROMOTER BINDING PROTEIN-LIKE TF, SPL9, was recently identified as a negative regulator of anthocyanin synthesis (Gou et al., 2011). Furthermore, three members of the Lateral Boundary Domain TF family, LBD37, LBD38, and LBD39, have been identified to directly repress expression of *PAP1* and *PAP2* which encode key regulators of anthocyanin biosynthesis in *A. thaliana* (Rubin et al., 2009).

Production of anthocyanin pigments during adverse growth conditions is considered one of the adaptive responses employed by plants (Chalker-Scott, 1999; Stewart et al., 2001; Peng et al., 2008; Cui et al., 2014) and again, interplay between positive and negative transcriptional regulators governs anthocyanin accumulation under unfavorable conditions. For example, under HL stress, expression of genes encoding the TFs that form the MBW complex, such as PAP1, PAP2, TT8, EGL3, TTG1 are strongly induced (Cominelli et al., 2008). Similar changes in the expression pattern of *PAP1, PAP2* and *TT8* were observed in *Arabidopsis* plants grown under different abiotic stress conditions (Kilian et al., 2007; Peng et al., 2008). In contrast, expression of negative regulators of anthocyanin biosynthesis such as *AtMYBL2, LBD37* and *LBD38* is reduced during stress conditions (Kilian et al., 2007; Dubos et al., 2008; Rubin et al., 2009). Sugars have also been implicated in the induction of anthocyanin pigments (Weiss, 2000; Teng et al., 2005; Solfanelli et al., 2006; Kilian et al., 2007; Dubos et al., 2008; Rubin et al., 2009), especially in the hormonal regulation of anthocyanin biosynthesis. For example, treatment of *Arabidopsis* with ABA and jasmonic acid (JA) induced anthocyanin production only in the presence of sucrose (Loreti et al., 2008). The involvement of sugars in the hormone-induced anthocyanin production seems relevant since the ABA and JA biosynthesis is triggered by salinity, drought and HL stresses (Wang et al., 2001; Walia et al., 2006; Galvez-Valdivieso et al., 2009; Ramel et al., 2013), and these adverse conditions also result in the increased accumulation of soluble sugar in different plant species (Lichtenthaler et al., 1981; Dubey and Singh, 1999; Kempa et al., 2008; Krasensky and Jonak, 2012; Schmitz et al., 2014).

In *Arabidopsis thaliana*, the NAC (No Apical Meristem/NAM, *Arabidopsis* ATAF1/2, and Cup-shaped Cotyledon2/CUC2) TF family has been shown to regulate diverse biological processes ranging from plant development to responses to stress conditions (Duval et al., 2002; Guo and Gan, 2006; Ko et al., 2007; Balazadeh et al., 2010; Yang et al., 2011; Lee et al., 2012; Mahmood et al., 2016). Although, some members of the NAC TF family have been implicated in modulating the phenylpropanoid pathway specific to lignin biosynthesis (Kubo et al., 2005; Mitsuda et al., 2005, 2007; Zhong et al., 2008; Ohashi-Ito et al., 2010), only a few have been identified to regulate anthocyanin biosynthesis. For instance, ANAC078 has been shown to positively regulate anthocyanin production under HL stress (Morishita et al., 2009) whereas JUB1/ANAC042 was shown to negatively affect the biosynthesis of anthocyanins in *Arabidopsis* (Wu et al., 2012). Here, we analyzed the role of ANAC032 in the regulation of anthocyanin biosynthesis. By employing biochemical, molecular and transgenic approaches, we show that ANAC032 negatively regulates anthocyanin biosynthesis in response to abiotic stresses including HL, salinity and oxidative stress. *ANAC032* expression was found to be induced by a range of stress conditions, suggesting an important regulatory role of ANAC032 in the biosynthesis of anthocyanin during stress.

## Materials and Methods

### Growth Conditions and Treatments

For sucrose-induced anthocyanin accumulation, surface sterilized seeds of wild-type and ANAC032 transgenic lines were germinated and grown on half-strength MS ager plates supplemented with 0, 1.5, 3, and 6% sucrose for 5 days under long-day (16 h/8 h light/dark) as well as under continuous light conditions. For JA-induced anthocyanin accumulation, surface sterilized seeds were germinated and grown on half-strength MS agar plates (1% sucrose) supplemented with 0, 25, and 50 μM MeJA for 12 days under long-day conditions (∼100 μmol m^-2^ s^-1^). For ABA treatment, 8-day-old *in vitro* grown seedlings were transferred to half-strength agar plates (1% sucrose) supplemented with 0, 10, and 20 μM ABA, and grown for 4 days under long-day conditions.

For HL stress, transgenic and wild-type plants were grown on LA4 Sunshine mix for 17 days under long-day growth conditions at 150 μmol m^-2^ s^-1^. Plants were then transferred to a separate growth chamber set at 450 μmol m^-2^ s^-1^ for 4 days. Plants grown at 150 μmol m^-2^ s^-1^ were treated as control (normal light, NL). For salinity stress, plants were irrigated with saline water (200 mM NaCl) three times at 4 days interval. Plants were harvested 2 weeks after the first irrigation.

### Generation of Transgenic Plants

Generation of transgenic plants, ANAC032 overexpression (35S:ANAC032), chimeric repressors (35S:ANAC032-SRDX) and P_ANAC032_:GUS, have been described in Mahmood et al. (2016).

### Histochemical Detection of B-glucuronidase (GUS) Activity

To analyze the GUS activity in response to HL stress, 20-day-old plants of the P_ANAC032_:GUS transgenic line were treated with optimal (control, 150 μmol m^-2^ s^-1^) or HL stress (HL, ∼1000 μmol m^-2^ s^-1^) for 24 h. The whole rosettes were first collected in chilled 90% acetone and were then immersed in GUS staining solution [100 mM sodium phosphate buffer pH 7.2, 10 mM EDTA, 0.5 mM potassium ferricyanide, 0.5 mM potassium ferrocyanide, 2 mM X-Gluc (5-bromo-4-chloro-3-indolyl-β-D-glucuronic acid), 0.1% triton X-100, 20% methanol]. The samples were incubated overnight in the dark at 37°C after a brief vacuum infiltration. Rosettes were photographed after removing chlorophyll using 70% ethanol. For GUS activity in response to sucrose treatment, seeds of the P_ANAC032_:GUS transgenic line were germinated and grown on half-strength MS agar plates supplemented with or without 1% sucrose for 5 days. The seedlings were subsequently transferred to fresh half-strength MS agar plates containing 1 and 6% sucrose for 24 h. After the treatment, seedlings were transferred to GUS staining solution and staining was performed as described above done in the same way as described for HL stress.

### Determination of Anthocyanin Content

Anthocyanin content was determined using the acidified methanol method with some modifications. For salinity and HL stress, ∼ 50 mg leaf tissues were homogenized in 500 μL of methanol-HCl (1% v/v) and kept at 4°C overnight. Samples were centrifuged at 14,000 rpm for 10 min. Supernatants were transferred to 2 mL centrifuge tubes. Anthocyanins were extracted using the extraction solvent chloroform:H_2_O 1.75:1 (v/v). Briefly, to each supernatant sample, chloroform and deionized H_2_O were added in 1.75:1 ratio (v/v) and vortexed vigorously for 30 s. Each sample was again centrifuged at 14,000 rpm for 10 min and the supernatant layer was transferred to a new 1.5 ml centrifuge tube. Samples were analyzed by spectrophotometric method at 530 and 657 nm wavelengths. Anthocyanin content was calculated by subtracting A_530_ values from those for A_657_. For sucrose- and MeJA-induced anthocyanin accumulation, twenty seedlings from each genotype were pooled in a single replicate after 5 and 12 days of growth, respectively. For ABA-induced anthocyanin, eight seedlings were pooled together in a single replicate after 4 days of treatment. For these three treatments, samples were homogenized in 250 μL of methanol-HCl (1% v/v) and anthocyanins were analyzed as described.

### Determination of Total Soluble Sugars

Total soluble sugars were determined through colorimetric quantification method as described in (Buysse and Merckx, 1993), with slight modifications. Briefly, 50 mg of the leaf tissues were homogenized in 0.5 mL ethanol (80%) and then centrifuged at 14,000 rpm for 5 min at 4°C. Supernatants were transferred to fresh 2 mL tubes. Homogenization and centrifugation steps were repeated 2–3 times, and supernatants were pooled. The final reaction mixture contained 0.5 mL of supernatants, 0.5 mL phenol (28% w/w) and 2.5 mL conc. sulphuric acid. Reaction was allowed to take place for 15 min and then the absorbance of the samples was recorded at 490 nm using plate reader. Glucose was used as a standard to determine the total soluble sugars in the samples.

### RNA Isolation, cDNA Synthesis and Quantitative RT-PCR Analysis

Total RNA from leaf samples was isolated using the Total RNA Purification Kit (Norgen Biotek Corp). One microgram of the total RNA from each sample was reverse transcribed into cDNA using qScript^TM^ cDNA SuperMix (Quanta Biosciences) according to the manufacturer’s protocol. Real-time RT-PCR was performed using the PerfeCTa^®^ SYBR^®^ Green SuperMix (Quanta Biosciences) following the manufacturer’s protocol. Expression values from two technical and three biological replicates were analyzed using the comparative CT method. *ACT7* and *UBC21* were used as internal reference genes for normalization. The primer sequences used in this study are provided in Supplementary Table S1.

### Histochemical Staining for Lignin Analysis (Wiesner Staining)

For lignin analysis in roots, 1-week-old seedlings were incubated in Wiesner solution (Phloroglucinol-HCl) (Mitra and Loqué, 2014) for 5 min and then visualized under a light microscope and photographed.

## Results

### ANAC032 Represses Sucrose-Induced Anthocyanin Biosynthesis

In a previous study, by generating and analyzing ANAC032 overexpressors (35S:ANAC032) and chimeric repressors (35S:ANAC032-SRDX), we have shown that ANAC032 promotes age-dependent and stress-induced senescence in *A. thaliana* (Mahmood et al., 2016). In addition to regulating the senescence processes, ANAC032 transgenic lines were also found to accumulate differential levels of anthocyanin pigments particularly during early growth phases (Supplementary Figure S1). Since production of anthocyanins is considered an important adaptive response particularly under stress conditions, we decided to investigate in more detail the mechanism by which ANAC032 affects anthocyanin accumulation during stress conditions.

**FIGURE 1:**
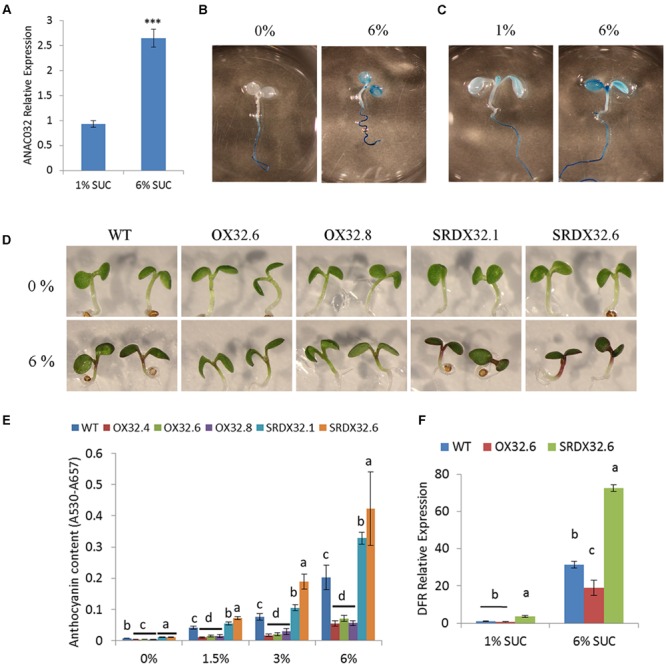
**Regulation of sucrose–induced anthocyanin by ANAC032 in *Arabidopsis*. (A)** qRT-PCR analysis of *ANAC032* expression in response to sucrose treatment. Five-day-old seedlings were transferred to fresh half-strength MS plates supplemented with 1% or 6% sucrose for 24 h under continuous light conditions. Data represent mean values (±SD). *UBC21* was used as internal control. Data were analyzed using Student’s *t*-test (^∗∗∗^*P*< 0.01). **(B,C)** β-glucuronidase activity of P_ANAC032_: GUS line in response to high sucrose treatment. Five-day-old seedlings of P_ANAC032_: GUS line, grown on half-strength MS plates with 0% **(B)** and 1% **(C)** sucrose were transferred to MS plates supplemented with 6% sucrose for 24 h, followed by incubation in GUS-staining solution overnight in dark at 37°C. **(D)** Phenotypic response of wild-type (WT) and ANAC032 transgenic lines (OX32; overexpressors and SRDX32; chimeric repressors) after 5 days of growth on half-strength agar plates containing 0 and 6% sucrose. **(E)** Anthocyanin content in the seedlings of ANAC032 transgenic lines, grown on half-strength MS agar plates containing 0, 1.5, 3, and 6% sucrose, after 5 days of growth. Data represent mean values (±SD; *n* = 3). **(F)** qRT-PCR analysis of *DFR* (dihydroflavanol reductase) expression in response to high sucrose treatment. Five-day-old seedlings of WT and ANAC032 transgenic lines were transferred to fresh half strength MS plates containing 1 and 6% sucrose for 24 h. *UBC21* was used as internal control. Within each treatment, bars with different letters in **(E,F)** are statistically not similar to each other according to one way ANOVA LSD test (*P* < 0.05).

Because sucrose is known to have a positive effect on the biosynthesis of anthocyanins, high sucrose treatment has been used as a reliable assay to analyze mutants involved in anthocyanin biosynthesis and regulation (Teng et al., 2005; Solfanelli et al., 2006; Rubin et al., 2009). To determine the role of ANAC032 in regulating anthocyanin biosynthesis, we first examined if the expression of *ANAC032* is affected by sucrose. qRT-PCR results showed that treatment of *Arabidopsis* seedlings with higher sucrose levels (6% sucrose) induced the expression of *ANAC032* compared to control conditions (1% sucrose) (**Figure 1A**). The effect of sucrose on the expression of *ANAC032* was further investigated in detail using a P_ANAC032_:GUS transgenic line. Histochemical analysis showed that the *ANAC032* promoter drove expression of *GUS* only in the roots when seedlings were grown on plates lacking sucrose. However, upon transfer to plates with 6% sucrose, an increased GUS activity was also detected in cotyledons (**Figure 1B**). A similar pattern of GUS activity was observed when P_ANAC032_:GUS seedlings were transferred from plates containing 1% sucrose to 6% sucrose (**Figure 1C**). A weak GUS activity was detected in the cotyledons of the P_ANAC032_:GUS seedlings grown on 1% sucrose, however, an obvious increase in the GUS activity was observed in the cotyledons upon transfer to 6% sucrose (**Figure 1C**). These results show that ANAC032 expression is induced by sucrose treatment, most likely in a concentration dependent manner.

Induction of *ANAC032* by sucrose prompted us to examine if anthocyanin biosynthesis may be altered in ANAC032 overexpression and SRDX lines upon treatment with sucrose. Seeds of wild-type and ANAC032 transgenic lines were grown on half-strength MS plates containing 0, 1.5, 3, and 6% sucrose under long-day growth conditions. After 5 days, anthocyanin levels increased in wild-type seedlings in a concentration dependent manner upon treatment with sucrose (**Figures 1C,D**). However, the seedlings of ANAC032 overexpression lines accumulated significantly lower anthocyanin pigments compared to wild-type seedlings (**Figures 1C,D**). In contrast, seedlings of both SRDX lines accumulated higher levels of anthocyanin pigments, suggesting that ANAC032 negatively regulates anthocyanin biosynthesis in *A. thaliana* in response to high sucrose (**Figures 1C,D**). The effect of high sucrose treatment on anthocyanin biosynthesis was further investigated by analyzing the expression of dihydroflavonol 4-reductase (DFR; responsible for the conversion of dihydroquercetin to leucocyanidin) in wild-type and transgenic lines. qRT-PCR results show that although *DFR* expression was induced by high sucrose in wild-type seedlings, its expression was significantly lower in an ANAC032 overexpression line (**Figure 1F**). On the contrary, an ANAC032 SRDX line had significantly higher transcript levels of *DFR* (**Figure 1F**).

### ANAC032 Represses Anthocyanin Production in Response to High Light Stress

**FIGURE 2:**
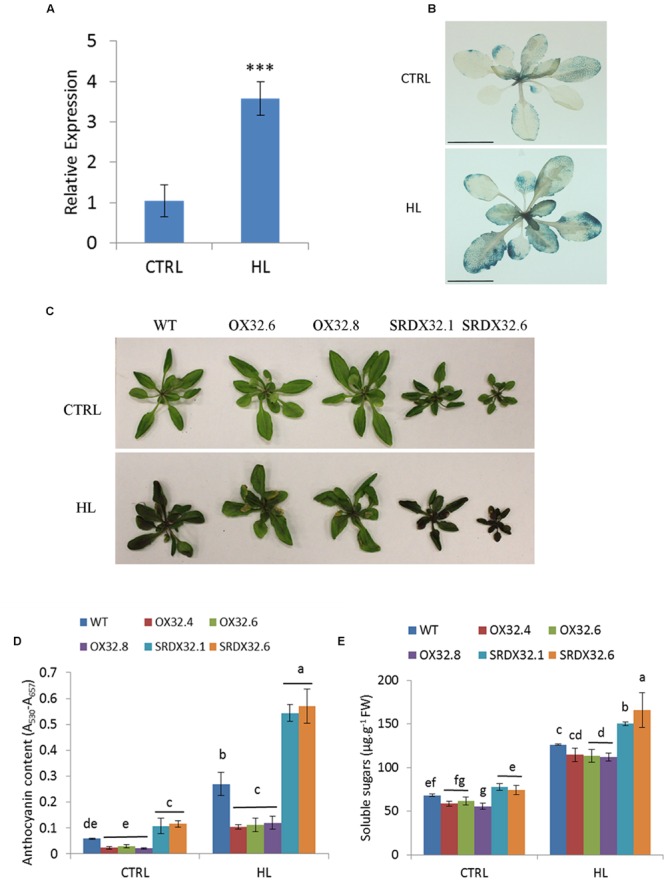
**Regulation of high light-induced anthocyanin biosynthesis. (A)** Expression analysis of *ANAC032* in response to high light stress using qRT-PCR assay. Data represent mean values (±SD). *ACT7* was used as internal control. Data were analyzed statistically using Student’s *t*-test (^∗∗∗^*P* < 0.01). **(B)** β-glucuronidase activity of P_ANAC032_: GUS line in response to high light treatment. Twenty-day-old plants of P_ANAC032_ : GUS line were transferred to control/optimal (150 μmol m^-2^ s^-1^) and high light (∼1000 μmol m^-2^ s^-1^) for 24 h and then incubated in GUS-staining solution overnight (scale bar = 1 cm). **(C)** Phenotype of WT and ANAC032 transgenic lines grown under optimal (control) and high light. **(D)** Biochemical analysis of anthocyanin content in WT and ANAC032 transgenic lines in response to high light stress. **(E)** Analysis of total soluble sugars in response to HL stress. 17-day-old plants of WT and ANAC032 overexpression and SRDX lines were grown under high light (∼450 μmol m^-2^ s^-1^) for 4 days. Plants grown under optimal light conditions (∼150 μmol m^-2^ s^-1^) were considered as control. Data represent values from three biological replicates. Bars with different letters are not statistically similar to each other according to one way ANOVA LSD test (*P* < 0.05).

In our previous study, ANAC032 was shown to promote leaf senescence in response to HL stress (Mahmood et al., 2016). Here, using qRT-PCR and P_ANAC032_:GUS line, we show that the expression of ANAC032 is induced by HL stress (**Figures 2A,B**). Analysis of ANAC032 transgenic lines exposed to a moderate HL stress (∼450 μmol m^-2^ s^-1^) showed that ANAC032 overexpression lines accumulated considerably lower levels of anthocyanin pigment compared to wild-type plants after 4 days of HL treatment (**Figures 2C,D**). In contrast, ANAC032 SRDX lines exhibited the opposite phenotype (**Figures 2C,D**). These results indicate that ANAC032 represses anthocyanin biosynthesis in response to HL stress. Since HL induces the accumulation of soluble sugars (Schmitz et al., 2014) and because ANAC032 transgenic lines accumulated altered anthocyanin content, we predicted that total soluble sugars accumulation might have been altered as well in the transgenic lines during HL stress. Indeed, ANAC032 SRDX lines accumulated significantly higher soluble sugars compared to wild-type under HL stress and at least two of the overexpression lines accumulated less (**Figure 2E**), suggesting a relationship between sugar metabolism and anthocyanin production.

**FIGURE 3:**
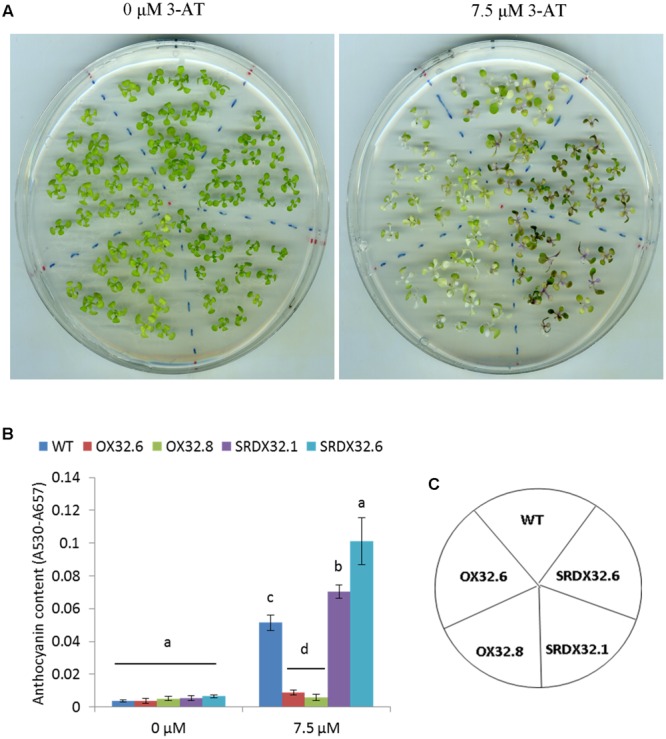
**Effect of 3-AT on anthocyanin biosynthesis in WT and ANAC032 transgenic lines. (A)** Phenotypic response of WT and ANAC032 overexpression and SRDX lines for anthocyanin biosynthesis in response to 3-AT treatment after 17 days of growth under long-day conditions. **(B)** Biochemical analysis of anthocyanin content in WT and ANAC032 transgenic lines after 17 days on 0 and 7.5 μM of 3-AT. **(C)** Template showing the position of the genotypes shown in **(A)**. Data represent mean values from three biological replicates. Within each treatment, bars with different letters are not statistically similar to each other according to one way ANOVA LSD test (*P* < 0.05).

Since HL stress leads to an oxidative burst associated with an increased proliferation of reaction oxygen species (ROS), we recapitulated the similar trend for anthocyanin biosynthesis in ANAC032 transgenic lines by exposing them to 3-AT. 3-AT is known to inhibit catalase activity, thereby resulting in an enhanced accumulation of H_2_O_2_. After 17 days of growth on half-strength MS agar plates supplemented with 0 and 7.5 μM 3-AT, ANAC032 overexpression lines accumulated considerably less anthocyanins compared to wild-type at 7.5 μM 3-AT (**Figures 3A,B**). On the contrary, ANAC032 SRDX lines accumulated significantly more anthocyanin pigments than wild-type (**Figures 3A,B**).

### Expression of Anthocyanin Biosynthesis and Regulatory Genes Is Affected by ANAC032

**FIGURE 4:**
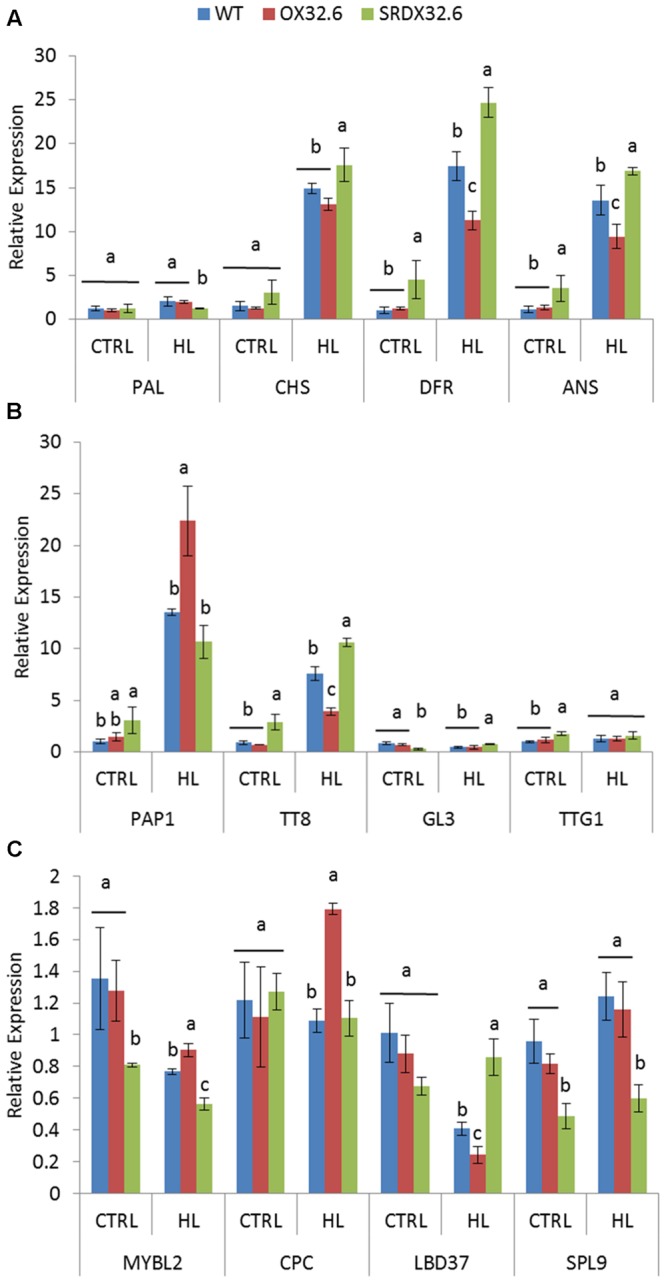
**Expression analysis of anthocyanin biosynthesis and regulatory genes in response to high light stress. (A)** qRT-PCR analysis of anthocyanin biosynthesis genes (ABGs). **(B)** qRT-PCR analysis of positive regulators (transcriptional activators) of anthocyanin biosynthesis. **(C)** qRT-PCR analysis of negative regulators (transcriptional repressors) of anthocyanin biosynthesis. Seventeen-day-old WT and ANAC032 transgenic lines were treated with optimal/control (150 μmol m^-2^ s^-1^) and high light (∼450 μmol m^-2^ s^-1^) for 4 days. Data represent mean relative expression values (±SD). *ACT7* was used as internal reference gene. Within each treatment, bars with different letters are not statistically similar to each other according one way ANOVA LSD test (*P* < 0.05).

Given the altered HL-induced anthocyanin accumulation in ANAC032 transgenic lines, we analyzed the molecular basis of these differences by investigating the transcript levels of genes involved in anthocyanin biosynthesis and its regulation. In the context of anthocyanin biosynthesis genes (ABGs), the expression levels of *PAL1, CHS, DFR* and *ANS* were determined in rosette leaves of wild-type and ANAC032 transgenic lines treated with HL stress. Consistent with the biochemical phenotype, qRT-PCR results showed that expression of particularly *CHS, DFR* and *ANS*, were drastically induced in wild-type leaves upon exposure to HL (**Figure 4A**). Interestingly, the expressions levels of both *DFR* and *ANS*, two late biosynthesis genes (LBGs), were significantly lower in the overexpression line compared to wild-type (**Figure 4A**) while transcript levels of both genes were significantly higher in the ANAC032 SRDX line (**Figure 4A**). Although expression of *CHS* was significantly induced in both transgenic lines, only the SRDX line presented significantly higher transcript levels compared to wild-type (**Figure 4A**). Next, we examined the transcript levels of regulatory genes known to activate or repress ABGs in *A. thaliana*. Amongst the activators, expression of *PAP1* (a MYB TF) and *TT8* (bHLH TF) was largely affected by HL in wild-type plants (**Figure 4B**). Unexpectedly, despite overexpression lines having significantly lower anthocyanin content (**Figure 3C**), *PAP1* transcript levels were higher in the overexpression line compared to wild-type under HL (**Figure 4B**). Although, the *PAP1* expression was higher in the SRDX line under control conditions, its expression was similar to wild-type under HL (**Figure 4B**). In the case of *TT8*, its transcript levels were significantly lower in the overexpression line compared to wild-type plants whereas they were higher in the SRDX line (**Figure 4B**). The expression of *GL3* (a bHLH homolog of *TT8*) and *TTG1* (WD40 repeat) were either slightly altered or remained unchanged in ANAC032 transgenic lines under both control and HL conditions (**Figure 4B**). In addition, we also examined the transcript abundance of known repressors of anthocyanin biosynthesis. Results show that expression of *MYBL2* and *LBD37* were downregulated by HL in wild-type plants whereas that of *CPC* and *SPL9* remained unchanged (**Figure 4C**). Under control conditions, the SRDX line displayed lower transcript levels of *MYBL2* while the overexpression line had transcript levels comparable to wild-type. However, under HL stress, the overexpression line and the SRDX line presented, respectively, higher and lower transcript levels of *MYBL2* than wild-type (**Figure 4C**). Transcript levels of *SPL9* were significantly reduced in the SRDX line but were similar in the overexpression line and wild-type under both control and HL conditions (**Figure 4C**). Although expression of *LBD37* were reduced in wild-type plants by HL treatment, its expression was further reduced in the overexpression line and induced in the SRDX line compared to the control condition (**Figure 4C**). The expression of *CPC* was only elevated in the overexpression line while remained unaltered in the SRDX line compared to wild-type plants under HL (**Figure 4C**).

### ANAC032 Negatively Regulates MeJA and ABA Signaling Required for Stress-Induced Anthocyanin Accumulation

**FIGURE 5:**
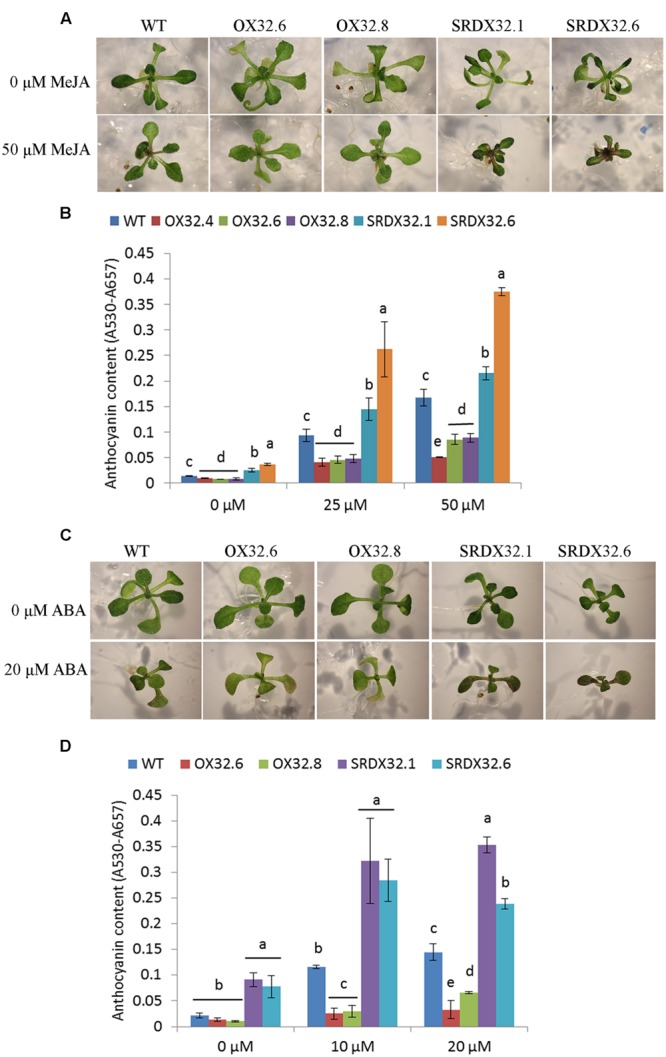
**Hormonal regulation of anthocyanin accumulation. (A)** Phenotypic response of WT and ANAC032 transgenic lines for anthocyanin biosynthesis in response to MeJA treatment after 15 days of growth under long-day conditions. **(B)** Anthocyanin content in response to MeJA treatment. Seeds were germinated and grown on half-strength MS agar plates (1% sucrose) supplemented with 0, 25, and 50 μM MeJA for 12 days under long-day conditions. Twenty seedlings were pooled in each replicate for each genotype to analyze anthocyanin content. **(C)** Phenotypic response of WT and ANAC032 transgenic lines for anthocyanin biosynthesis in response to ABA treatment. **(D)** Anthocyanin content in response to ABA treatment. Eight-day-old seedlings of wild-type and ANAC032 transgenic lines were exposed to 0, 10, and 20 μM ABA and were grown under long-day condition for 4 days. Eight seedlings were pooled in each replicate for each genotype. Data represent mean values (±SD, *n* = 3). Within each treatment, bars with different letters in **(B,D)** are not similar to each other statistically according to one way ANOVA LSD test (*P* < 0.05).

Stress-regulated plant hormones such as ABA and JA are also known to induce anthocyanin biosynthesis in plants, suggesting that stresses can induce anthocyanin accumulation through signals integrated by these stress hormones (Loreti et al., 2008; Shan et al., 2009). Since expression of ANAC032 is induced by MeJA and ABA (Mahmood et al., 2016), we hypothesized that ANAC032 may inhibit JA- or ABA-inductive effects on anthocyanin production. First, we studied the effect of MeJA on anthocyanin biosynthesis. Results show that after 12 days of growth on plates supplemented with 0, 25, and 50 μM MeJA, wild-type seedlings accumulated anthocyanins in a concentration dependent manner (**Figures 5A,B**). The effect of MeJA on the accumulation of anthocyanins was reduced in ANAC032 overexpression lines compared to wild-type (**Figures 5A,B**). In contrast, ANAC032 SRDX lines accumulated significantly higher levels of anthocyanin pigments compared to wild-type seedlings (**Figures 5A,B**). Similar findings were observed when ANAC032 transgenic lines were treated with ABA (**Figures 5C,D**). Taken together these results suggest that ANAC032 negatively regulates ABA and JA signals that lead to anthocyanin accumulation.

### ANAC032 Negatively Regulates Anthocyanin Biosynthesis in Response to Salinity Stress

**FIGURE 6:**
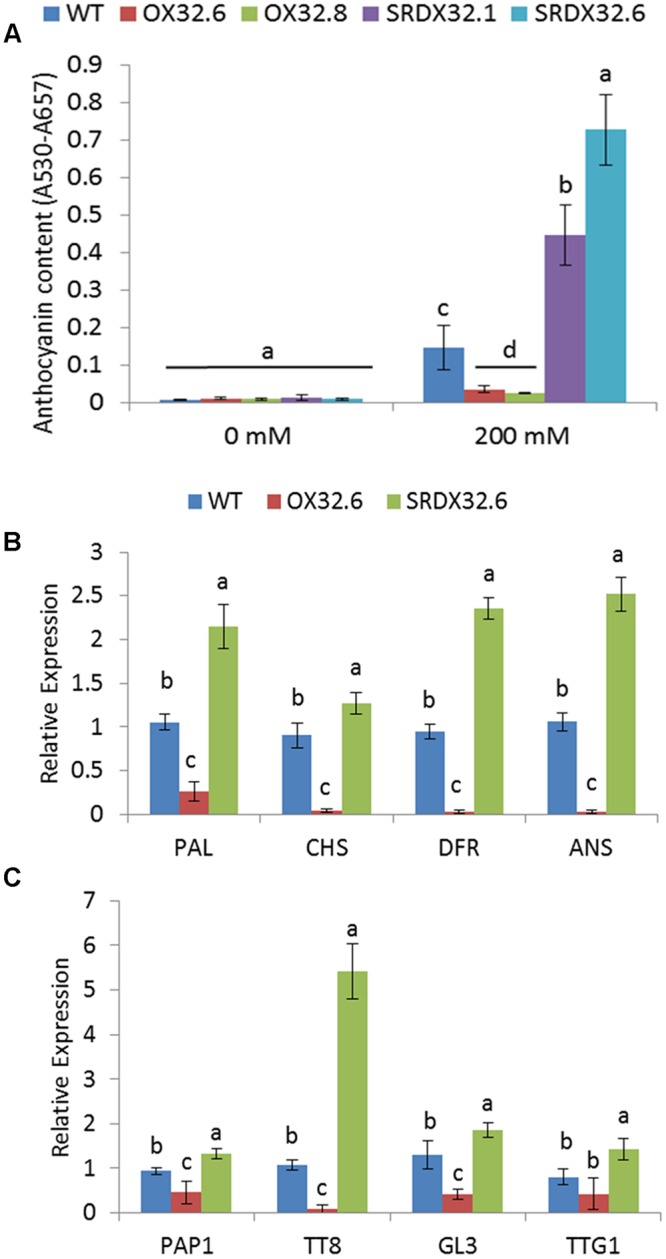
**Salinity-induced anthocyanin biosynthesis in ANAC032 transgenic lines. (A)** Anthocyanin content in response to salinity stress. Three-week-old plants of WT and ANAC032 transgenic lines were treated with 200 mM NaCl for 2 weeks. Data represent values from three biological replicates (±SD). Bars with same letters are not significantly different from each other according to one way ANOVA LSD test (*P* < 0.05). **(B)** qRT-PCR analysis of ABGs in leaves of plants subjected to salinity stress. **(C)** qRT-PCR analysis of transcription factors that positively regulate the expression of ABGs plants subjected to salinity stress. *ACT7* was used as internal control. For each gene, bars with same letters are not significantly different from each other according to one way ANOVA LSD test (*P* < 0.05).

Given the inhibitory effect of ANAC032 on the ABA- and MeJA-induced anthocyanin accumulation, we speculated that ANAC032 would also negatively regulate anthocyanin production in response to stresses that trigger ABA and JA biosynthesis, such as salinity stress (Zhu, 2002; Walia et al., 2006). Consistent with this, challenging WT and ANAC032 transgenic lines with 200 mM saline solutions (NaCl) showed that ANAC032 overexpression lines accumulated less anthocyanin contents whereas the SRDX lines accumulate more compared to wild-type, suggesting that ANAC032 can also reduce anthocyanin production in response to salinity stress (**Figure 6A**). The altered accumulation of anthocyanin pigment in ANAC032 transgenic lines was further confirmed by analyzing the expression of anthocyanin biosynthesis and regulatory genes. Since no significant differences in anthocyanin content were found between genotypes under control conditions, the expression analysis was only performed on plants exposed to salinity stress. Consistent with these biochemical changes, transcript levels of all the ABGs analyzed (*PAL, CHS, DFR* and *ANS*) were significantly reduced in the overexpression line compared to wild-type (**Figure 6B**) and the opposite expression pattern in the SRDX line, particularly for *DFR* and *ANS* (**Figure 6B**). In accordance with the reduced transcript levels of these biosynthesis genes, expression of *PAP1, TT8* and *GL3*, which constitute the transcriptional complex to activate the expression of ABGs, was also drastically reduced in the overexpression line compared to wild-type (**Figure 6C**). The expression of these genes, including *TTG1*, in contrast, was significantly induced in the SRDX line (**Figure 6C**). Similar to what was observed in the case of HL stress; the most drastic differences were observed for *TT8* in the ANAC032 transgenic lines (**Figure 6C**).

### Lignin Biosynthesis Is Not Regulated by ANAC032

**FIGURE 7:**
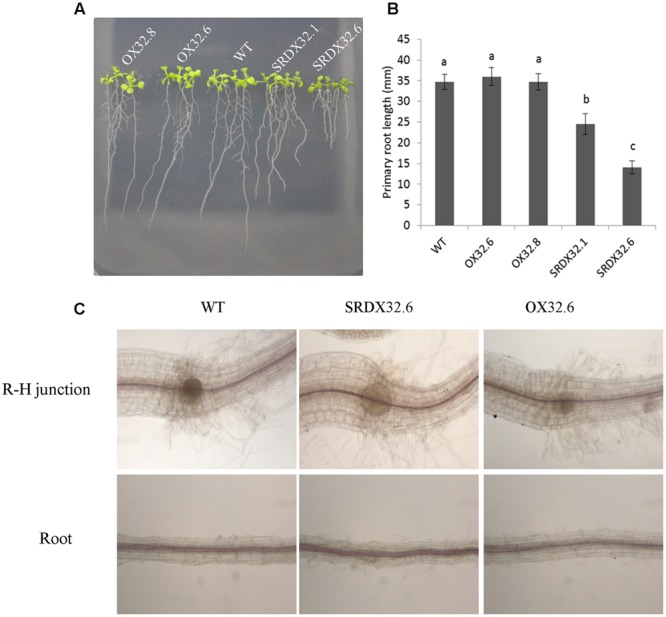
**(A)** Phenotype of 10-day-old seedlings grown on half-strength MS agar plates supplemented with 1% sucrose under long-day condition. **(B)** Primary root lengths of 7-day-old seedlings of wild-type and ANAC032 transgenic lines. Data represent values from three biological replicates. Each replicate included 20 roots. Bars with same letters are not statistically different from each other according to one way ANOVA-LSD test (*P* < 0.05). **(C)** Analysis of lignification pattern in WT and ANAC032 transgenic lines. Phloroglucinol-HCl staining of lignin in roots and hypocotyl regions of 7-day-old seedlings. Roots were analyzed under a light microscope (10 X magnification). (R-H, root-hypocotyl junction).

ANAC032 is predominantly expressed in roots under normal growth conditions (**Figures 1B,C**), suggesting that its expression may influence root development. Analysis of primary root growth showed that although both wild-type and ANAC032 overexpression lines had comparable primary root growth, the chimeric repressors produced significantly shorter roots under normal growth conditions (**Figures 7A,B**). Since lignin and anthocyanin are produced from same phenylpropanoid pathway and many mutants exhibiting ectopic lignification produce shorter roots (Abdulrazzak et al., 2006; Lucas et al., 2011) we anticipated that ANAC032 may affect lignin biosynthesis in the roots. Phloroglucinol-HCL staining of roots showed that both ANAC032 overexpression and SRDX lines had lignification patterns similar to wild-type as no ectopic lignification was observed in any of the transgenic line (**Figure 7C**). These results show that ANAC032 is unlikely involved in the regulation of lignin biosynthesis, and that the retarded root growth in SRDX032 lines could be due to reduced cell division and/or expansion.

## Discussion

In plants, spatio-temporal regulation of anthocyanin biosynthesis takes place in a highly ordered manner (Martins et al., 2013; Xu et al., 2013). A marked increase in the accumulation of anthocyanin pigment has been observed during a variety of biotic and abiotic stresses and they have been proposed to have a protective role during stress conditions (Chalker-Scott, 1999). While the enzymatic pathway involved in the production of anthocyanins is largely known, the transcriptional regulation of their biosynthetic pathway is not fully understood. In this study, we investigated the role of ANAC032 in the regulation of anthocyanin production in *A. thaliana*.

A number of NAC TFs have been implicated in mediating stress responses in *A. thaliana*, yet only JUB1 and ANAC078 have been implicated in the regulation of anthocyanin production (Morishita et al., 2009; Wu et al., 2012). An increase in the expression of late ABGs (e.g., *DFR* and *LDOX/ANS*) was observed in transgenic plants overexpressing *ANAC078*. Similarly, TT2, TT8 and TTG1 that form the MBW complex were also induced in the ANAC078 lines (Morishita et al., 2009). *JUB1* expression, however, was shown to have a negative impact on the expression of ABGS and MBWs in *Arabidopsis* (Wu et al., 2012). Although, *JUB1* was shown to be important for survival under stress conditions, such as salinity and oxidative stress (Wu et al., 2012), the regulation of anthocyanin biosynthesis has not been studied in detail under these stress conditions. We show that in early growth stages, ANAC032 had a negative impact on production of anthocyanin pigment which was lost in the later stage of plant growth (Supplementary Figure S1). However, under different stress conditions such as HL, oxidative stress and salinity stress, there is a clear negative effect on the production of anthocyanin pigments in plants overexpressing *ANAC032* (**Figures 2**, **3**, and **6**). At the molecular level, we also observed a reduction in the transcript levels of late ABGs (such as *DFR* and *LDOX/ANS*) in an ANAC032 overexpression line and induction of those genes in the ANAC032-SRDX line compared to wild-type (**Figure 4A**). In the context of TFs forming the MBW complex and that regulate the expression of ABGs, the expression of *TT8* was significantly reduced in the overexpression line and induced in the SRDX line compared to wild-type, suggesting that a negative impact on anthocyanin biosynthesis may be due to a reduction in *TT8* expression which eventually affects the assembly of the MBW complex (**Figure 4B**). TT8 activates the expression of *DFR* by directly binding with its promoter region (Baudry et al., 2006). Despite reduced accumulation of anthocyanin pigments, the ANAC032 overexpression line had significantly higher transcript levels of *PAP1* compared to wild-type, yet they accumulate less anthocyanin. Given the reduced transcript levels of *TT8* in overexpression plants under HL, it is likely that increased *PAP1* expression is a response to lower levels of *TT8* to compensate for its deficiency. This proposition is in agreement with previous findings proposing that PAP1 and its MYB homolog, TT2, can activate the expression of *TT8* (Baudry et al., 2006).

Studies have shown that *TT8* is mainly regulated at the transcriptional level, and that TT8 can regulate its own expression (Nesi et al., 2000; Baudry et al., 2006). Furthermore, new mechanisms for the negative transcriptional regulation of *TT8* and *DFR* have also been elucidated with the identification of repressors of anthocyanin biosynthesis. For example, AtMYBL2 has been shown to interact with TT8 (Matsui et al., 2008) along with GL3 and EGL3 (Dubos et al., 2008) resulting in the formation of an inactive complex, L2BW, which competes with the active MBW complex to regulate ABGs (Dubos et al., 2008; Matsui et al., 2008). Similarly, SPL9, another repressor of anthocyanin biosynthesis, interferes with the formation of the MBW complex by competing with TT8 for its interaction with PAP1 (Gou et al., 2011). Since ANAC032 overexpressors and chimeric repressors exhibited contrasting phenotypes with regard to anthocyanin accumulation, it is highly unlikely that ANAC032 directly represses the expression of *DFR* and *TT8*. The repressive effect on the expression of these genes, therefore, appears to be indirect which could be due to another factor that represses the activation of these genes. Consistent with this is that ANAC032-SRDX plants had reduced expression of *AtMYBL2* under both control and HL whereas the overexpression line presents increased transcription of *AtMYBL2* compared to wild-type, particularly under HL stress (**Figure 4C**). The altered transcript levels of *AtMYBL2* may, therefore, be responsible for the differential expressions of *TT8* and *DFR* in the ANAC032 transgenic lines under HL stress. Moreover, the expression of *SPL9* was only reduced in the ANAC032-SRDX line under both control and HL conditions. The three members of the *Lateral Organ Boundary Domain family* of TF, LBD37, LBD38 and LBD39, negatively regulate anthocyanin biosynthesis in response to HL, high sucrose and nitrogen stress conditions in *A. thaliana* (Rubin et al., 2009). In our study, the expression of *LBD37* was repressed by HL in wild-type plants (**Figure 4C**), however, it was more drastically reduced in the overexpression line. Since these TFs directly repress the expression of *PAP1/2* (Rubin et al., 2009), a significant increase in the expression of *PAP1* was observed in the overexpression line under HL stress compared to wild-type. Despite the obvious induction of *PAP1* in overexpression line, it was unable to overcome the negative impact on the expression of TT8 and DFR in ANAC032 overexpression line under HL.

Most abiotic stresses have been shown to cause the accumulation of soluble sugars (glucose, fructose, and sucrose) in several plant species (Kempa et al., 2008; Krasensky and Jonak, 2012). In grape skin, production of anthocyanin has also been correlated with sugar accumulation (Boss et al., 1996). Moreover, exogenous sucrose treatment triggers the induction of anthocyanin production in *Arabidopsis* (Teng et al., 2005; Solfanelli et al., 2006). Hyper-accumulation of soluble sugars during stress conditions, therefore, may be not only important for acclimation responses but may also trigger anthocyanin biosynthesis. Consistent with this hypothesis is that the *suc2* mutant which lacks a functional phloem-loading Suc-proton symporter-, not only accumulates increased anthocyanin pigments but also contains higher levels of soluble sugars (Lloyd and Zakhleniuk, 2004). Similarly, another *Arabidopsis* mutant, *phosphoglucomutase (pgm)*, which accumulates soluble sugars, had higher levels of anthocyanin pigments compared to wild-type and had a drastic increase in the expression of *PAP1, 4CL, CHS, CHI, F3H, DFR*, and *LDOX* (Solfanelli et al., 2006). We show that *ANAC032* is also induced by sucrose treatment and altering *ANAC032* expression negatively regulated anthocyanin production upon treatment with exogenous sucrose in a concentration dependent manner (**Figure 1**). Based on these findings, it is likely that ANAC032 represses anthocyanin production by altering sugar metabolism. In agreement with this, is that the ANAC032 overexpression lines, which accumulate less anthocyanin, had reduced levels of soluble sugars and that the SRDX032 lines accumulated more pigments compared to wild-type, particularly under HL.

A number of studies have shown that plants produce hormones like ABA and JA when exposed to different abiotic stress conditions such as HL, drought and salinity (Wang et al., 2001; Walia et al., 2006; Galvez-Valdivieso et al., 2009; Ramel et al., 2013). Production of these stress hormones evokes various adaptive responses in plants, including anthocyanin production. We have shown that *ANAC032* expression is also induced by ABA and MeJA treatment. Similar to what was observed under HL and salinity stress, ANAC032 overexpression lines accumulated significantly less anthocyanins whereas SRDX lines accumulated more pigments upon ABA and MeJA treatment compared to wild-type (**Figure 5**). These findings suggest that ANAC032 represses anthocyanin biosynthesis through a negative regulation of ABA and JA signaling. The altered anthocyanin production in response to ABA and MeJA may also involve differential accumulation of soluble sugars as was observed in the case of HL stress. Several lines of evidence support this notion; (i) the ABA and MeJA-induced anthocyanin biosynthesis in *A. thaliana* was shown to be dependent upon the presence of sucrose in the nutrient medium (Loreti et al., 2008); (ii) many ABA-, JA- and stress-inducible genes are also co-regulated by sugars (Reinbothe et al., 1994; Sadka et al., 1994); and (iii) exogenous treatment with ABA results in the accumulation of soluble sugars, thus mimicking a stress response (Hiratsuka et al., 2001; Nagao et al., 2005).

In summary, we have investigated the role of ANAC032 in the regulation of anthocyanin biosynthesis in *A. thaliana*. Induced expression of *ANAC032* represses anthocyanin accumulation and alters the expression of anthocyanin biosynthesis (*DFR, ANS/LDOX*) and regulatory genes (*TT8*) under stresses, more likely through modulated expression of the negative regulators of anthocyanin biosynthesis, *AtMYBL2* and *SPL9*. However, given that the identification of the complete network of negative regulators of anthocyanin biosynthesis in *Arabidopsis* is not fully known, modulation of yet other negative regulators by ANAC032 can not be ruled out. Our data also suggest that the differential accumulation of anthocyanin is due to altered ABA, MeJA and sugar signaling.

## Author Contributions

KM and SR conceived the project. KM, AE-K, JC, and SR designed the experiments. KM and ZX performed the experiments. KM analyzed the data. KM, JC, and SR wrote the manuscript. All authors read and approved the final version of the manuscript.

## Conflict of Interest Statement

The authors declare that the research was conducted in the absence of any commercial or financial relationships that could be construed as a potential conflict of interest.

## Abbreviations

3-AT: 3-aminotriazole
ABA: abscisic acid
*ATAF1,2* and *CUC2*: (cup-shaped cotyledon)
GUS: β-glucuronidase
HL: high light
MeJA: methyl jasmonate
NAC: *NAM* (no apical meristem)

## References

[B1] Abdulrazzak N. Pollet B. Ehlting J. Larsen K. Asnaghi C. Ronseau S. (2006). A coumaroyl-ester-3-hydroxylase insertion mutant reveals the existence of nonredundant meta-hydroxylation pathways and essential roles for phenolic precursors in cell expansion and plant growth. *Plant Physiol.* 140 30–48. 10.1104/pp.105.069690 16377748 PMC1326029

[B2] Albert N. W. Lewis D. H. Zhang H. Irving L. J. Jameson P. E. Davies K. M. (2009). Light-induced vegetative anthocyanin pigmentation in *Petunia*. *J. Exp. Bot.* 60 2191–2202. 10.1093/jxb/erp097 19380423 PMC2682507

[B3] Balazadeh S. Siddiqui H. Allu A. D. Matallana-Ramirez L. P. Caldana C. Mehrnia M. (2010). A gene regulatory network controlled by the NAC transcription factor ANAC092/AtNAC2/ORE1 during salt-promoted senescence. *Plant J.* 62 250–264. 10.1111/j.1365-313X.2010.04151.x 20113437

[B4] Baudry A. Caboche M. Lepiniec L. (2006). TT8 controls its own expression in a feedback regulation involving TTG1 and homologous MYB and bHLH factors, allowing a strong and cell-specific accumulation of flavonoids in *Arabidopsis thaliana*. *Plant J.* 46 768–779. 10.1111/j.1365-313X.2006.02733.x 16709193

[B5] Boss P. K. Davies C. Robinson S. P. (1996). Anthocyanin composition and anthocyanin pathway gene expression in grapevine sports differing in berry skin colour. *Aust. J. Grape Wine Res.* 2 163–170. 10.1111/j.1755-0238.1996.tb00104.x

[B6] Buysse J. A. N. Merckx R. (1993). An improved colorimetric method to quantify sugar content of plant tissue. *J. Exp. Bot.* 44 1627–1629. 10.1093/jxb/44.10.1627

[B7] Carey C. C. Strahle J. T. Selinger D. A. Chandler V. L. (2004). Mutations in the pale aleurone color1 regulatory gene of the *Zea mays* anthocyanin pathway have distinct phenotypes relative to the functionally similar TRANSPARENT TESTA GLABRA1 gene in *Arabidopsis thaliana*. *Plant Cell* 16 450–464. 10.1105/tpc.018796.1 14742877 PMC341916

[B8] Castellarin S. D. Pfeiffer A. Sivilotti P. Degan M. Peterlunger E. Di Gaspero G. (2007). Transcriptional regulation of anthocyanin biosynthesis in ripening fruits of grapevine under seasonal water deficit. *Plant Cell Environ.* 30 1381–1399. 10.1111/j.1365-3040.2007.01716.x 17897409

[B9] Chalker-Scott L. (1999). Environmental significance of anthocyanins in plant stress responses. *Photochem. Photobiol.* 70 1–9. 10.1111/j.1751-1097.1999.tb01944.x

[B10] Christie P. J. Alfenito M. R. Walbot V. (1994). Impact of low-temperature stress on general phenylpropanoid and anthocyanin pathways: enhancement of transcript abundance and anthocyanin pigmentation in maize seedlings. *Planta* 194 541–549. 10.1007/BF00714468

[B11] Cominelli E. Gusmaroli G. Allegra D. Galbiati M. Wade H. K. Jenkins G. I. (2008). Expression analysis of anthocyanin regulatory genes in response to different light qualities in *Arabidopsis thaliana*. *J. Plant Physiol.* 165 886–894. 10.1016/j.jplph.2007.06.010 17766004

[B12] Cui L. G. Shan J. X. Shi M. Gao J. P. Lin H. X. (2014). The miR156-SPL9-DFR pathway coordinates the relationship between development and abiotic stress tolerance in plants. *Plant J.* 80 1108–1117. 10.1111/tpj.12712 25345491

[B13] Dubey R. S. Singh A. K. (1999). Salinity induces accumulation of soluble sugars and alters the activity of sugar metabolising enzymes in rice plants. *Biol. Plant.* 42 233–239. 10.1023/A:1002160618700

[B14] Dubos C. Le Gourrierec J. Baudry A. Huep G. Lanet E. Debeaujon I. (2008). MYBL2 is a new regulator of flavonoid biosynthesis in *Arabidopsis thaliana*. *Plant J.* 55 940–953. 10.1111/j.1365-313X.2008.03564.x 18532978

[B15] Duval M. Hsieh T. F. Kim S. Y. Thomas T. L. (2002). Molecular characterization of AtNAM: a member of the *Arabidopsis* NAC domain superfamily. *Plant Mol. Biol.* 50 237–248. 10.1023/A:1016028530943 12175016

[B16] Eryilmaz F. (2006). The relationships between salt stress and anthocyanin content in higher plants. *Biotechnol. Biotechnol. Equip.* 20 47–52. 10.1080/13102818.2006.10817303

[B17] Galvez-Valdivieso G. Fryer M. J. Lawson T. Slattery K. Truman W. Smirnoff N. (2009). The high light response in *Arabidopsis* involves ABA signaling between vascular and bundle sheath cells. *Plant Cell* 21 2143–2162. 10.1105/tpc.108.061507 19638476 PMC2729609

[B18] Gonzalez A. Zhao M. Leavitt J. M. Lloyd A. M. (2008). Regulation of the anthocyanin biosynthetic pathway by the TTG1/bHLH/Myb transcriptional complex in *Arabidopsis* seedlings. *Plant J.* 53 814–827. 10.1111/j.1365-313X.2007.03373.x 18036197

[B19] Gou J. Felippes F. F. Liu C. Weigel D. Wang J. (2011). Negative regulation of anthocyanin biosynthesis in *Arabidopsis* by a miR156-targeted SPL transcription factor. *Plant Cell* 23 1512–1522. 10.1105/tpc.111.084525 21487097 PMC3101539

[B20] Guo Y. Gan S. (2006). AtNAP, a NAC family transcription factor, has an important role in leaf senescence. *Plant J.* 46 601–612. 10.1111/j.1365-313X.2006.02723.x 16640597

[B21] Hiratsuka S. Onodera H. Kawai Y. Kubo T. Itoh H. Wada R. (2001). ABA and sugar effects on anthocyanin formation in grape berry cultured in vitro. *Sci. Hortic.* 90 121–130. 10.1016/S0304-4238(00)00264-8

[B22] Hughes N. M. Neufeld H. S. Burkey K. O. (2005). Functional role of anthocyanins in high-light winter leaves of the evergreen herb *Galax urceolata*. *New Phytol.* 168 575–587. 10.1111/j.1469-8137.2005.01546.x 16313641

[B23] Jackson D. Roberts K. Martin C. (1992). Temporal and spatial control of expression of anthocyanin biosynthetic genes in developing flowers of *Antirrhinum majus*. *Plant J.* 2 425–434. 10.1111/j.1365-313X.1992.00425.x

[B24] Jiang C. Gao X. Liao L. Harberd N. P. Fu X. (2007). Phosphate starvation root architecture and anthocyanin accumulation responses are modulated by the gibberellin-DELLA signaling pathway in *Arabidopsis*. *Plant Physiol.* 145 1460–1470. 10.1104/pp.107.103788 17932308 PMC2151698

[B25] Kempa S. Krasensky J. Dal Santo S. Kopka J. Jonak C. (2008). A central role of abscisic acid in stress-regulated carbohydrate metabolism. *PLoS ONE* 3:e3935 10.1371/journal.pone.0003935 PMC2593778 19081841

[B26] Kilian J. Whitehead D. Horak J. Wanke D. Weinl S. Batistic O. (2007). The AtGenExpress global stress expression data set: protocols, evaluation and model data analysis of UV-B light, drought and cold stress responses. *Plant J.* 50 347–363. 10.1111/j.1365-313X.2007.03052.x 17376166

[B27] Ko J.-H. Yang S. H. Park A. H. Lerouxel O. Han K.-H. (2007). ANAC012, a member of the plant-specific NAC transcription factor family, negatively regulates xylary fiber development in *Arabidopsis thaliana*. *Plant J.* 50 1035–1048. 10.1111/j.1365-313X.2007.03109.x 17565617

[B28] Krasensky J. Jonak C. (2012). Drought, salt, and temperature stress-induced metabolic rearrangements and regulatory networks. *J. Exp. Bot.* 63 1593–1608. 10.1093/jxb/err460 22291134 PMC4359903

[B29] Kubo M. Udagawa M. Nishikubo N. Horiguchi G. Yamaguchi M. Ito J. (2005). Transcription switches for protoxylem and metaxylem vessel formation. *Genes Dev.* 19 1855–1860. 10.1101/gad.1331305 16103214 PMC1186185

[B30] Lee S. Seo P. J. Lee H.-J. Park C.-M. (2012). A NAC transcription factor NTL4 promotes reactive oxygen species production during drought-induced leaf senescence in *Arabidopsis*. *Plant J.* 70 831–844. 10.1111/j.1365-313X.2012.04932.x 22313226

[B31] Lichtenthaler H. K. Buschmann C. Doll M. Fietz H. J. Bach T. Kozel U. (1981). Photosynthetic activity, chloroplast ultrastructure, and leaf characteristics of high-light and low-light plants and of sun and shade leaves. *Photosynth. Res.* 2 115–141. 10.1007/BF00028752 24470202

[B32] Lloyd J. C. Zakhleniuk O. V. (2004). Responses of primary and secondary metabolism to sugar accumulation revealed by microarray expression analysis of the *Arabidopsis* mutant, pho3. *J. Exp. Bot.* 55 1221–1230. 10.1093/jxb/erh143 15133053

[B33] Loreti E. Povero G. Novi G. Solfanelli C. Alpi A. Perata P. (2008). Gibberellins, jasmonate and abscisic acid modulate the sucrose-induced expression of anthocyanin biosynthetic genes in *Arabidopsis*. *New Phytol.* 179 1004–1016. 10.1111/j.1469-8137.2008.02511.x 18537890

[B34] Lotkowska M. E. Tohge T. Fernie A. R. Xue G.-P. Balazadeh S. Mueller-Roeber B. (2015). The *Arabidopsis* transcription factor MYB112 promotes anthocyanin formation during salinity and under high light stress. *Plant Physiol.* 169 1862–1880. 10.1104/pp.15.00605 26378103 PMC4634054

[B35] Lucas M. Swarup R. Paponov I. A. Swarup K. Casimiro I. Lake D. (2011). SHORT-ROOT regulates primary, lateral, and adventitious root development in *Arabidopsis*. *Plant Physiol.* 155 384–398. 10.1104/pp.110.165126 21030506 PMC3075784

[B36] Mahmood K. El-Kereamy A. Kim S.-H. Nambara E. Rothstein S. J. (2016). ANAC032 positively regulates age-dependent and stress-induced senescence in *Arabidopsis thaliana*. *Plant Cell Physiol.* 10.1093/pcp/pcw120 [Epub ahead of print]. 27388337

[B37] Martins T. R. Berg J. J. Blinka S. Rausher M. D. Baum D. A. (2013). Precise spatio-temporal regulation of the anthocyanin biosynthetic pathway leads to petal spot formation in *Clarkia gracilis* (Onagraceae). *New Phytol.* 197 958–969. 10.1111/nph.12062 23231386 PMC3540125

[B38] Matsui K. Umemura Y. Ohme-Takagi M. (2008). AtMYBL 2, a protein with a single MYB domain, acts as a negative regulator of anthocyanin biosynthesis in *Arabidopsis*. *Plant J.* 55 954–967. 10.1111/j.1365-313X.2008.03565.x 18532977

[B39] Mitra P. P. Loqué D. (2014). Histochemical staining of *Arabidopsis thaliana* secondary cell wall elements. *J. Vis. Exp.* 87:e51381 10.3791/51381 PMC4186213 24894795

[B40] Mitsuda N. Iwase A. Yamamoto H. Yoshida M. Seki M. Shinozaki K. (2007). NAC transcription factors, NST1 and NST 3, are key regulators of the formation of secondary walls in woody tissues of *Arabidopsis*. *Plant Cell* 19 270–280. 10.1105/tpc.106.047043 17237351 PMC1820955

[B41] Mitsuda N. Seki M. Shinozaki K. Ohme-takagi M. (2005). The NAC transcription factors NST1 and NST2 of *Arabidopsis* regulate secondary wall thickenings and are required for anther dehiscence. *Plant Cell* 17 2993–3006. 10.1105/tpc.105.036004.1 16214898 PMC1276025

[B42] Morishita T. Kojima Y. Maruta T. Nishizawa-Yokoi A. Yabuta Y. Shigeoka S. (2009). *Arabidopsis* NAC transcription factor, ANAC 078, regulates flavonoid biosynthesis under high-light. *Plant Cell Physiol.* 50 2210–2222. 10.1093/pcp/pcp159 19887540

[B43] Nagao M. Minami A. Arakawa K. Fujikawa S. Takezawa D. (2005). Rapid degradation of starch in chloroplasts and concomitant accumulation of soluble sugars associated with ABA-induced freezing tolerance in the moss *Physcomitrella patens*. *J. Plant Physiol.* 162 169–180. 10.1016/j.jplph.2004.06.012 15779827

[B44] Nakabayashi R. Yonekura-Sakakibara K. Urano K. Suzuki M. Yamada Y. Nishizawa T. (2014). Enhancement of oxidative and drought tolerance in *Arabidopsis* by overaccumulation of antioxidant flavonoids. *Plant J.* 7 367–379. 10.1111/tpj.12388 PMC4282528 24274116

[B45] Nemie-feyissa D. Olafsdottir S. M. Heidari B. Lillo C. (2014). Phytochemistry nitrogen depletion and small R3-MYB transcription factors affecting anthocyanin accumulation in *Arabidopsis* leaves. *Phytochemistry* 98 34–40. 10.1016/j.phytochem.2013.12.006 24388610

[B46] Nesi N. Debeaujon I. Jond C. Pelletier G. Caboche M. Lepiniec L. (2000). The TT8 gene encodes a basic helix-loop-helix domain protein required for expression of DFR and BAN genes in *Arabidopsis* siliques. *Plant Cell* 12 1863–1878. 10.1105/tpc.12.10.1863 11041882 PMC149125

[B47] Ohashi-Ito K. Oda Y. Fukuda H. (2010). *Arabidopsis* VASCULAR-RELATED NAC-DOMAIN6 directly regulates the genes that govern programmed cell death and secondary wall formation during xylem differentiation. *Plant Cell* 22 3461–3473. 10.1105/tpc.110.075036 20952636 PMC2990123

[B48] Peng M. Hudson D. Schofield A. Tsao R. Yang R. Gu H. (2008). Adaptation of *Arabidopsis* to nitrogen limitation involves induction of anthocyanin synthesis which is controlled by the NLA gene. *J. Exp. Bot.* 59 2933–2944. 10.1093/jxb/ern148 18552353 PMC2504352

[B49] Procissi A. Dolfini S. Ronchi A. Tonelli C. (1997). Light-dependent spatial and temporal expression of pigment regulatory genes in developing maize seeds. *Plant Cell* 9 1547–1557. 10.1105/tpc.9.9.1547 12237395 PMC157032

[B50] Quattrocchio F. Verweij W. Kroon A. Spelt C. Mol J. Koes R. (2006). PH4 of *Petunia* is an R2R3 MYB protein that activates vacuolar acidification through interactions with basic-helix-loop-helix transcription factors of the anthocyanin pathway. *Plant Cell* 18 1274–1291. 10.1105/tpc.105.034041 16603655 PMC1456866

[B51] Ramel F. Ksas B. Havaux M. (2013). Jasmonate: a decision maker between cell death and acclimation in the response of plants to singlet oxygen. *Plant Signal. Behav.* 8:e26655 10.4161/psb.26655 PMC4091353 24103864

[B52] Reinbothe S. Mollenhauer B. Reinbothe C. (1994). JIPs and RIPs: the regulation of plant gene expression by jasmonates in response to environmental cues and pathogens. *Plant Cell* 6 1197–1209. 10.1105/tpc.6.9.1197 7919988 PMC160513

[B53] Rubin G. Tohge T. Matsuda F. Saito K. Scheible W.-R. (2009). Members of the LBD family of transcription factors repress anthocyanin synthesis and affect additional nitrogen responses in *Arabidopsis*. *Plant Cell* 21 3567–3584. 10.1105/tpc.109.067041 19933203 PMC2798321

[B54] Sadka A. DeWald D. B. May G. D. Park W. D. Mullet J. E. (1994). Phosphate modulates transcription of soybean VspB and other sugar-inducible genes. *Plant Cell* 6 737–749. 10.2307/3869876 12244255 PMC160472

[B55] Schmitz J. Heinrichs L. Scossa F. Fernie A. R. Oelze M. L. Dietz K. J. (2014). The essential role of sugar metabolism in the acclimation response of *Arabidopsis thaliana* to high light intensities. *J. Exp. Bot.* 65 1619–1636. 10.1093/jxb/eru027 24523502 PMC3967092

[B56] Shan X. Zhang Y. Peng W. Wang Z. Xie D. (2009). Molecular mechanism for jasmonate-induction of anthocyanin accumulation in *Arabidopsis*. *J. Exp. Bot.* 60 3849–3860. 10.1093/jxb/erp223 19596700

[B57] Solfanelli C. Solfanelli C. Poggi A. Poggi A. Loreti E. Loreti E. (2006). Sucrose-specic induction of the anthocyanin biosynthetic pathway in *Arabidopsis*. *Plant Physiol.* 140 637–646. 10.1104/pp.105.072579 16384906 PMC1361330

[B58] Stewart A. J. Chapman W. Jenkins G. I. Graham I. Martin T. Crozier A. (2001). The effect of nitrogen and phosphorus deficiency on flavonol accumulation in plant tissues. *Plant, Cell Environ.* 24 1189–1197. 10.1046/j.1365-3040.2001.00768.x

[B59] Teng S. Keurentjes J. Bentsink L. Koornneef M. Smeekens S. (2005). Sucrose-specific induction of anthocyanin biosynthesis in *Arabidopsis* requires the MYB75/PAP1 gene. *Plant Physiol.* 139 1840–1852. 10.1104/pp.105.066688.1840 16299184 PMC1310563

[B60] Vanderauwera S. Zimmermann P. Rombauts S. Vandenabeele S. Langebartels C. Gruissem W. (2005). Genome-wide analysis of hydrogen peroxide-regulated gene expression in *Arabidopsis* reveals a high light-induced transcriptional cluster involved in anthocyanin biosynthesis. *Plant Physiol.* 139 806–821. 10.1104/pp.105.065896 16183842 PMC1255997

[B61] Viola I. L. Camoirano A. Gonzalez D. H. (2016). Redox-dependent modulation of anthocyanin biosynthesis by the TCP transcription factor TCP15 during exposure to high light intensity conditions in *Arabidopsis*. *Plant Physiol.* 170 74–85. 10.1104/pp.15.01016 26574599 PMC4704573

[B62] Walia H. Wilson C. Wahid A. Condamine P. Cui X. Close T. J. (2006). Expression analysis of barley (*Hordeum vulgare* L.) during salinity stress. *Funct. Integr. Genomics* 6 143–156. 10.1007/s10142-005-0013-0 16450154

[B63] Walker A. R. Davison P. A. Bolognesi-Winfield A. C. James C. M. Srinivasan N. Blundell T. L. (1999). The TRANSPARENT TESTA GLABRA1 locus, which regulates trichome differentiation and anthocyanin biosynthesis in *Arabidopsis*, encodes a WD40 repeat protein. *Plant Cell* 11 1337–1349. 10.1105/tpc.11.7.1337 10402433 PMC144274

[B64] Wang X. Q. Ullah H. Jones A. M. Assmann S. M. (2001). G protein regulation of ion channels and abscisic acid signaling in *Arabidopsis* guard cells. *Science* 292 2070–2072. 10.1126/science.1059046 11408655

[B65] Weiss D. (2000). Regulation of flower pigmentation and growth: multiple signaling pathways control anthocyanin synthesis in expanding petals. *Physiol. Plant* 110 152–157. 10.1034/j.1399-3054.2000.110202.x

[B66] Wu A. Allu A. D. Garapati P. Siddiqui H. Dortay H. Zanor M.-I. (2012). JUNGBRUNNEN 1, a reactive oxygen species-responsive NAC transcription factor, regulates longevity in *Arabidopsis*. *Plant Cell* 24 482–506. 10.1105/tpc.111.090894 22345491 PMC3315228

[B67] Xu W. Grain D. Le Gourrierec J. Harscoët E. Berger A. Jauvion V. (2013). Regulation of flavonoid biosynthesis involves an unexpected complex transcriptional regulation of TT8 expression, in *Arabidopsis*. *New Phytol.* 198 59–70. 10.1111/nph.12142 23398515

[B68] Yamane T. Seok T. J. Goto-Yamamoto N. Koshita Y. Kobayashi S. (2006). Effects of temperature on anthocyanin biosynthesis in grape berry skins. *Am. J. Enol. Vitic.* 57 54–59.

[B69] Yang S.-D. Seo P. J. Yoon H.-K. Park C.-M. (2011). The *Arabidopsis* NAC transcription factor VNI2 integrates abscisic acid signals into leaf senescence via the COR/RD genes. *Plant Cell* 23 2155–2168. 10.1105/tpc.111.084913 21673078 PMC3160032

[B70] Zhong R. Lee C. Zhou J. McCarthy R. L. Ye Z. H. (2008). A battery of transcription factors involved in the regulation of secondary cell wall biosynthesis in *Arabidopsis*. *Plant Cell* 20 2763–2782. 10.1105/tpc.108.061325 18952777 PMC2590737

[B71] Zhu H. Fitzsimmons K. Khandelwal A. Kranz R. G. (2009). CPC, a single-repeat R3 MYB, is a negative regulator of anthocyanin biosynthesis in *Arabidopsis*. *Mol. Plant* 2 790–802. 10.1093/mp/ssp030 19825656

[B72] Zhu J. (2002). Salt and drought stress signal transduction in plants. *Annu. Rev. Plant Biol.* 53 247–273. 10.1146/annurev.phyto.40.120501.101443 12221975 PMC3128348

