# Supplementary material for: The Arabidopsis Transcription Factor ANAC032 Represses Anthocyanin Biosynthesis in Response to High Sucrose and Oxidative and Abiotic Stresses
Source: Front Plant Sci. 2016 Oct 14;7:1548. doi: 10.3389/fpls.2016.01548 (PMC5063858; doi:10.3389/fpls.2016.01548)
Supplement: Supplementary file 1 [file Presentation_1.PDF]

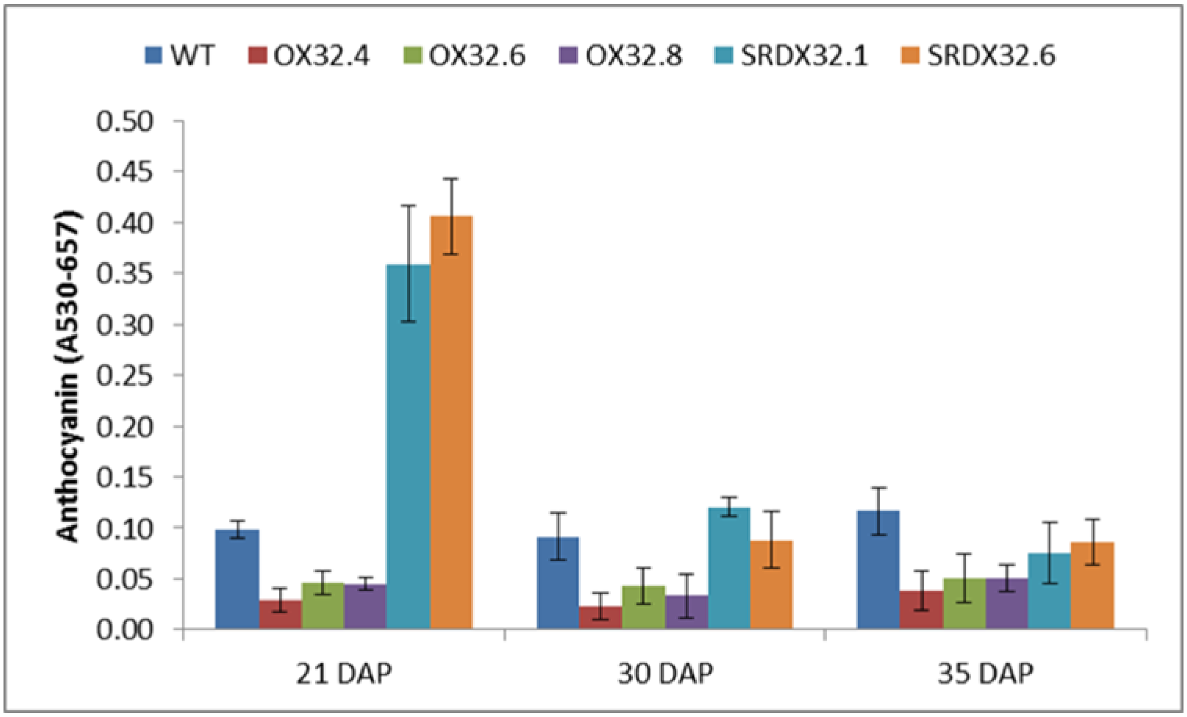


**Supplemental Figure. 1**: Analysis of anthocyanin content in ANAC032 transgenic lines.

Anthocyanin pigments were determined in the rosette leaves of wild-type and ANAC032 transgenic lines at 21, 30 and 35 DAP. Data represent mean values form three biological replicates (±SD).

| **Supplementary Table S1:** List of primers used in this study for quantitative RT-PCR analysis | |
|---|---|
| **Oligo Name** | **Oligo Sequence** |
| | **5' → 3'** |
| **qNAC32-F** | GTCGGTTGAGTAATGCCCTTGA |
| **qNAC32-R** | CCCAATAGCCGTGACACAATCT |
| **PAL-F** | ATTAACGGGGCACACAAGAG |
| **PAL-R** | GTCTCCGCCGCATAACATAG |
| **CHS-F** | GCGTGCGTTCTCTTCATACTAGA |
| **CHS-R** | GGCCACACCATCCTTAGC |
| **DFR-F** | AACGGATGTGACGGTGTTTT |
| **DFR-R** | TCCATTCACTGTCGGCTTTA |
| **ANS-F** | TTCTTTCATCTTGCGTATCC |
| **ANS-R** | ACTCACTCGTTGCTTCTAT |
| **PAP1-F** | GTATGGAGAAGGCAAATGG |
| **PAP1-R** | TGAAGGCGAAGAAGAAGA |
| **TT8-F** | AGAGCATCAGCAAGTGAA |
| **TT8-R** | GCGGTAGCCTCTTATCTT |
| **GL3-F** | TCTCATTCGGTTCAATCCT |
| **GL3-R** | ATTCCTGGTGTCGCTATT |
| **TTG1-F** | TGTTCAGTCCTCCTTCTC |
| **TTG1-R** | GCTCTACATCGTTCCAATC |
| **LBD37-F** | CGTCTTCGTCGCTAAATTCTTC |
| **LBD37-R** | GCAACGACTGAAACAAAGCA |
| **MYBL2-F** | GAAGACGATCTCATCCTCAAGC |
| **MYBL2-R** | AACTTCGTTGTCGGTTCGTC |
| **CPC-F** | AAGGCTTCTTGTTCCGAAGAG |
| **CPC-R** | CCTGTCGCCAACGAGTTTAT |
| **SPL9-F** | GACACCGAGTTTGTGGAGTG |
| **SPL9-R** | AATTCCGGAAGCTGATGAAA |
| **ACT7-F** | TGCACCGCCAGAGAGAAAAT |
| **ACT7-R** | TGAGGGATGCAAGGATTGATC |
| **UBC21-F** | TCCTCTTAACTGCGACTCAGG |
| **UBC21-R** | GCGAGGCGTGTATACATTTG |
